# Associations Between Quality of Nursing Work Life, Work Ability Index and Intention to Leave the Workplace and Profession: A Cross-Sectional Study Among Nurses in Croatia

**DOI:** 10.3390/ijerph22081192

**Published:** 2025-07-30

**Authors:** Snježana Čukljek, Janko Babić, Boris Ilić, Slađana Režić, Biljana Filipović, Jadranka Pavić, Ana Marija Švigir, Martina Smrekar

**Affiliations:** 1 Department of Nursing, University of Applied Health Sciences, 10000 Zagreb, Croatia; boris.ilic@zvu.hr (B.I.); sladjanarezic@gmail.com (S.R.); biljana.filipovic@zvu.hr (B.F.); jadranka.pavic@zvu.hr (J.P.); anamarija.svigir@zvu.hr (A.M.Š.); martina.smrekar@zvu.hr (M.S.); 2 Department of Nursing, Faculty of Health Studies, University of Rijeka, 51000 Rijeka, Croatia; 3 Department of Health Psychology, University of Applied Health Sciences, 10000 Zagreb, Croatia; janko.babic@zvu.hr; 4 University Hospital Centre Zagreb, 10000 Zagreb, Croatia

**Keywords:** nurse, work ability, quality of work life, intention to leave

## Abstract

Introduction: Nurses are the largest group of healthcare workers, and healthcare managers should pay attention to the quality of work life and the health and working capacity of nurses in order to ensure a sufficient number of nurses and a stable workforce. Aim: The present study aimed to determine nurses’ quality of work life, work ability index and intention to leave the nursing profession and to examine the associations between nurses’ quality of work life, work ability index and intention to leave the nursing profession. Methods: An online cross-sectional study was conducted. A total of 498 nurses completed the instrument, consisting of demographic data, Brooks’ Quality of Nursing Work Life Survey (BQNWL), Work Ability Index Questionnaire (WAIQ) and questions on their intention to leave their current job or the nursing profession. Results: Most nurses had a moderate quality of work life (QWL) (73.7%) and a good work ability index (WAI) (43.78%). Men (*p* = 0.047), nurses who study (*p* = 0.021), nurses who do not have children (*p* = 0.000) and nurses who do not take care of their parents (*p* = 0.000) have a statistically significantly higher total WAIQ score. Most nurses (61.1%) had considered changing jobs in the last 12 months, and 36.9% had considered leaving the nursing profession. A statistically significant positive correlation was found between the total BQNWL and the total WAI. The study found no correlation between QWL, WAI and intention to change jobs or leave the profession, which was unexpected. Conclusions: To ensure the provision of necessary nursing care and a healthy working environment for nurses, it is necessary to regularly monitor QWL and WAI and take measures to ensure the highest quality of working life. Further longitudinal and mixed-methods research is needed to understand the relationship between QWL, WAI and intention to leave.

## 1. Introduction

Nurses are the largest group of healthcare workers in the Republic of Croatia and make up more than half of the healthcare workforce. As in many other countries, there is a shortage of nurses. In addition, there is an aging population, shorter patient stays in healthcare facilities and faster patient turnover, increased care in day hospitals and an increase in the number of medical procedures performed [1], which increases the workload of nurses. For this reason, healthcare managers should pay attention to the quality of work life and the health and working capacity of nurses in order to ensure a sufficient number of nurses and a stable workforce.

Quality of nurses’ work life (QNWL) is “a degree to which registered nurses are able to satisfy important personal needs through their experiences in their organization while achieving the organization’s goals” [2], p. 323. QNWL is a subjective experience that is influenced by numerous factors related to the specifics of the workplace, as well as factors from one’s personal life. QNWL is a multidimensional construct that encompasses the physical, social, psychological and environmental dimensions of the employee. QNWL is associated with numerous positive outcomes, such as increased efficiency and productivity, organizational commitment and job satisfaction, lower turnover, less burnout, greater patient and nurse satisfaction, and higher quality of services provided [3,4,5,6]. On the other hand, in situations where QNWL is lower, there is a tendency to leave the job, to have lower job satisfaction and to have higher stress levels [7,8].

The most common factors contributing to lower QNWL are inadequate work schedule, poor staffing, lack of autonomy, high work stress, performance of non-nursing tasks, lack of opportunities for professional and personal development, inadequate work environment, poor nursing leadership style and inadequate salary [3,4,5,9,10].

Managers should pay attention to the QNWL of their nurses, as facilities with higher QNWL have higher staff retention and greater interest in employment [10], while facilities with lower quality QNWL experience absenteeism and a higher turnover rate among nursing staff [11]. If the facility focuses on improving quality of working life, it can increase staff satisfaction, loyalty and productivity, both individually and for the organization. Satisfied nurses are efficient, committed to their work and fulfill their obligations [12].

Work ability (WA) is an important concept in occupational health research [13] and is defined as an “individual’s capacity to match job demands according to his/her physical and mental conditions and work circumstances” [14]. As WA is multifactorial, it varies within an individual depending on position or working conditions [15]. The Work Ability Index Questionnaire (WAIQ) is the most widely used questionnaire for self-assessment of WA [16]. The results of previous studies have shown that the WAIQ is able to significantly predict sickness-related absenteeism, incapacity to work, retirement and mortality [17].

Poor WA increases job stress and has an impact on the quality of work life [18]. Evidence from the literature has confirmed that nurses’ quality of working life affects their job satisfaction and ultimately their intention to leave or keep their job [18]. According to Martinez et al. [19], impaired WA and early exit from the profession have a negative impact on the labor market, health and pension systems. Camerino et al. [20] demonstrated that low WA was associated with a higher intention to leave the nursing profession in a sample of registered nurses in 10 European countries. The results of recent research have shown a strong association between impaired WA and intention to leave the nursing profession. Several factors were common to both outcomes: high psychosocial risk for job strain, effort/reward imbalance, exposure to situations that may contribute to musculoskeletal pain/injury, and younger age. Overcommitment, lower job skills and sedentariness were associated with impaired WA, while insomnia symptoms and male gender were also associated with intention to leave the nursing profession. Of the sociodemographic factors examined, higher age was found to be protective against impaired WA and intention to leave the profession [19]. In a recent meta-analysis, the worldwide pooled prevalence of inadequate work ability among hospital nursing personnel was found to be 24.7% [15].

The intention to leave the profession is the process of thinking, planning and making a decision about leaving a job or a profession [21], but a person does not necessarily have to leave the profession. The results of a recently published survey in several European countries show that 8.4% of nurses intend to leave the hospital and 13.6% intend to leave the profession [22]. Job dissatisfaction, poor working conditions, inadequate salary, lack of opportunities for career development, nursing burnout, bullying, emotional distress, disappointment with the reality of nursing, lack of support, and mismatch between education and reality are factors that influence job change and leaving the profession [22,23,24,25,26].

During the COVID-19 pandemic, in addition to the factors mentioned above, having served on the front line against COVID-19, a lack of equipment, the need to care for children and early retirement also contributed to the intention to leave the profession [27].

Research shows that lower quality of life and lower work ability in nurses influence their intention to leave the profession, but the relationship between quality of work life, work ability and intention to leave the profession has not yet been explored. Given the shortage of nurses, heavy workloads, the aging of nurses in the Republic of Croatia and the perception of the nursing profession as difficult and poorly paid, it is necessary to determine the quality of working life, work ability index, intention to leave the profession and the connection between these constructs in order to plan interventions aimed at improving working conditions, retaining nurses in the profession and attracting new nurses to the profession.

The aim of this study is to determine nurses’ quality of work life, work ability index and intention to leave the nursing profession. Also, the study aimed to examine the associations between nurses’ quality of work life, work ability index, and intention to leave the nursing profession.

## 2. Materials and Methods

### 2.1. Study Design

An online cross-sectional study was conducted.

### 2.2. Participants

The participants in the study were nurses employed in hospitals and outpatient facilities in the Republic of Croatia. The snowball sampling technique was used. A total of 674 nurses started filling out the questionnaire, and 498 nurses filled out the questionnaire completely. Prior to conducting the study, the minimum required sample size was determined using a sample size calculator. Based on an estimated population of approximately 32,000 nurses in the Republic of Croatia, with a confidence level of 95% and a margin of error of 5%, the required minimum sample size was calculated to be 380 nurses. The final sample meets and exceeds the calculated minimum, ensuring the adequacy of the sample for statistical analysis. Nurses completed the questionnaire voluntarily and gave their consent to participate in the study. The snowball sampling technique was used to ensure that as many nurses as possible from different parts of the country participated in the study. The questionnaire was forwarded twice to an email address from the researchers’ personal address books with a request to forward the questionnaire to their colleagues. The criteria for inclusion in the study were being a registered nurse and currently working as a nurse. Exclusion criteria were being a nursing student, another healthcare professional or unemployed.

### 2.3. Data Collection

Data were collected in May and June 2023. A link to a web-based questionnaire (LimeSurvey platform of the University Computing Centre (SRCE) of the University of Zagreb) was emailed to nurses, asking them to complete the survey and forward the link to their colleagues. The nurses were sent a reminder to complete the survey one week after the first email. In the email and on the cover page of the questionnaire, the purpose of the survey was explained, and it was pointed out that participation in the survey was voluntary and that nurses could withdraw from completing the questionnaire at any time. Nurses had to confirm that they agreed to take part in the study, and only then could they start filling out the questionnaire. Prior to data analysis, participants with the same IP addresses were deleted. Also, participants who did not enter data regarding employment as a nurse were removed from further analysis.

### 2.4. Instruments

In the study, a four-part instrument was used. The first part contained demographic data, the second part was the Brooks’ Quality of Nursing Work Life Survey (BQNWL), the third part was the Work Ability Index Questionnaire (WAIQ) and the fourth part contained questions about the intention to leave the current job or the nursing profession.

The nurses provided the following demographic data: gender, age, marital status, having children, age of children, taking care of parents, attending a nursing study program, level, place of employment and years of service.

The Brooks Quality of Nursing Work Life Survey (BQNWL) contains 42 items in 4 subscales: work life–home life (7 items); work design (10 items); work context (20 items); and work world (5 items). The work life–home life subscale measures the interface between the life experiences of nurses in their place of work and at home. The work design subscale refers to the composition of nursing work and describes what nurses do. The work context subscale refers to the practice environments in which nurses work and examines the impact of the work environment on both nurses and patient systems. And the work world subscale measures the impact of broad societal influences and changes on the practice [28]. Nurses indicate their agreement with each statement on a scale of 1 to 6, where 1 indicates “strongly disagree” and 6 “strongly agree” [28]. On the BQNWL, nurses can score 45–252, where 42–112 is low QNWL, 113–182 is moderate QNWL and 183–252 is high QNWL. In Brooks’ research, Cronbach’s α was 0.89. Prior to its implementation, the BQNWL was translated into Croatian in accordance with the cross-cultural adaptation framework developed by Beaton et al. [29]. The translation and adaptation of the questionnaire were carried out by a group of nursing managers and nurses from an educational institution. Subsequently, an independent person (English teacher) translated it again into English, and the translations were compared. A pilot test was conducted with 20 participants (nursing students) who completed the pre-final version and stated ambiguities and doubts. The final version of the questionnaire was created and applied to the study. The internal consistency of the questionnaire was assessed using Cronbach’s alpha coefficient, and in our research a satisfactory result of α = 0.887 was obtained.

The Work Ability Index Questionnaire (WAIQ) was used to assess work ability (WA). It was developed by Finnish researchers based on the Finnish Longitudinal Study on Municipal Employees [17,29]. The WAIQ consists of 10 questions divided into 7 items: 1—current WA compared with the lifetime best; 2—WA in relation to job demands; 3—number of current diseases diagnosed by a physician; 4—estimated work impairment due to diseases; 5—sick leave in the past year; 6—personal prognosis of WA after 2 years; and 7—mental resources related to the worker’s life in general, both at work and during leisure time [29]. All questions are closed-ended questions with a different number of possible answers. The WAI is a summary measure obtained by summing up the scores of the individual responses to all items of the WAIQ and ranges from 7 to 49 points. A higher WAI indicates a better WA [29]. The WAI can be analyzed as a numerical value or categorized into four categories: 7–27 points for a poor WAI, 28–36 points for a moderate WAI, 37–43 points for a good WAI and 44–49 points for an excellent WAI [30]. The WAIQ has already been translated into Croatian (WAIQ-CRO), so we used the Croatian version of the WIAQ [31]. The internal consistency of the questionnaire was assessed using Cronbach’s alpha coefficient, and in our research a satisfactory result of α = 0.73 was obtained, indicating acceptable reliability.

Nurses were asked to answer whether they had thought about changing jobs or leaving the nursing profession in the last year and to state the reason why.

### 2.5. Data Analysis

Statistical analyses were carried out using IBM SPSS Statistics 25. Descriptive statistics were used to summarize and describe the sociodemographic characteristics of the nurses. Normality of variable distributions was assessed using the Kolmogorov–Smirnov test. Nonparametric tests, including the Mann–Whitney U test and the Kruskal–Wallis test, were used to examine differences between groups. In addition, Spearman’s rank-order correlation and point biserial correlation coefficients were calculated to examine the relationships between variables. A significance level of *p* < 0.05 was applied throughout the analyses.

### 2.6. Ethical Consideration

The study was approved by an institutional Ethics committee (Admin. No.: 602-03/22-18/621; ref. no.: 251-379-10-22-02). Participants were informed about the purpose of the study and informed consent was obtained. The principles of the Declaration of Helsinki were adhered to. Participants did not receive any incentives for participating in the research. Prior to conducting the research, written consent was obtained from the authors of the Brooks’ Quality of Nursing Work Life Survey and the Work Ability Index Questionnaire. 

## 3. Results

The study included 498 nurses, most of whom were women (*n* = 443; 88.96%), aged 41–50 years (*n* = 132; 26.5%) and married (*n* = 279; 56%). Most nurses had children (*n* = 322; 64.7%), and a quarter of the nurses were students (*n* = 125; 25.1%). The demographic data are presented in Table 1.

In our study, most nurses had a moderate QNWL (*n* = 331, 73.7%), and only eight nurses (1.8%) had a low QNWL (Table 2). The mean score was 165.26 (range 93–240 points), with the highest score in the work context domain (Table 3).

Most nurses had a good WAI (*n* = 218, 43.78%), and two-thirds of the nurses had a good or excellent WAI. Only 5.42% of the nurses had a poor WAI score (Table 2).

When analyzing the data related to QNWL, no statistically significant differences were found in relation to gender, age, study status, childcare or parental care.

Men (M-W U test = 9785.00; *p* = 0.047), nurses who are studying (M-W U test = 20,094.050; *p* = 0.021), nurses who do not have children (M-W U test = 34,461.00; *p* = 0.000) and nurses who do not care for their parents (M-W U test = 35,426.00; *p* = 0.000) have a statistically significant higher total score on the WAIQ.

Most nurses (*n* = 277, 61.1%) had considered changing jobs in the last 12 months, and more than a third of nurses (*n* = 167, 36.9%) had considered leaving the nursing profession (Table 4).

An intercorrelation analysis was conducted for age, length of service, BQNWL subscales, total BQNWL and total WAI. As far as age is concerned, the only significant correlation is with the total WAI—the younger nurses have a higher WAI and the nurses with a longer length of service have a lower WAI. This is in line with the results of the difference test, in which only age was a significant factor for differences in the WAI. There is also a statistically significant positive correlation between the total BQNWL and the total WAI (Table 5).

No statistically significant correlation was found between the nurses’ results on the WAI and BQNWL and the intention to leave the profession or the intention to change jobs.

A statistically significant correlation was found between nurses’ results on the WAI and BQNWL, as well as a statistically significant correlation between the results related to thoughts of changing jobs and leaving the nursing profession (Table 6).

## 4. Discussion

Our study has shown a correlation between the work ability index and the quality of nurses’ work life. Although these concepts are related and have an impact on nurses’ work, they are two distinct concepts. QNWL refers to the ability to meet personal needs while simultaneously fulfilling work tasks and achieving the desired goals of the institution, while WA refers to an individual’s capacity to match job demands according to physical and mental conditions and work circumstances.

In our study, the range of QNWL scores is 93–240, with a mean score of 165.26, indicating a moderate QNWL. The results for the majority of nurses in our study (73.7%) indicate a moderate QNWL. In most other studies, nurses also reported a moderate QNWL, with the mean ranging from 139.45 to 179.99 [7,28,32,33,34,35,36,37]. The highest average score was achieved in the study by Al Masakri et al. [34] (M = 179.99), which was conducted before the COVID-19 pandemic, which may have affected the results. Nurses in our study achieved higher scores compared to the studies by Salahat et al. [7], Suleiman et al. [33] and Rosahangar et al. [35], which were conducted in different cultural settings and with different perceptions of the nursing profession, which may have affected the mean average scores. In a previous study conducted in Croatia on 102 nurses, most of them also had a moderate QNWL, but the average result was higher, 172.2, and none of the nurses had a low QNWL [38]. The slightly lower average results in our study could be related to the fact that the study was conducted in more institutions and with a larger number of nurses.

In three areas of the questionnaire, namely, work life–home life, work design and work world, nurses reported a moderate QNWL. In the area of work context, however, the nurses reported a high QNWL. The work context subscale refers to the practice settings in which nurses work and examines the impact of the work environment on both nurses and patient systems. In relation to the above domain, nurses mostly agree with the statement that it is important for them to have a designated private break area for nursing staff, that the hospital offers degree-granting programs for nurses, and that they are able to communicate well with their nurse manager/supervisor. A quarter of the nurses are currently studying, so the possibility of receiving a scholarship is important to them.

Most nurses agree or completely agree that their work has an impact on their families. The nursing profession is dominated by women who, in addition to their professional role, also have to fulfill a number of family responsibilities, which include caring for children and sick family members. Shift work and on-call duties can further interfere with home life [39]. In our study, a relatively neutral result was observed with regard to the impact of shift work, which could be related to the fact that most nurses work rotating shifts, which allows more time for other activities and a better balance between work, family and other commitments compared to working in the morning shift. However, results from previous research indicate the adverse impact of shift work, which leads to poor nurse well-being and reduced opportunities for social support [40,41], longer sick leave [42], fatigue and sleep disorders [41], and an increased risk of obesity, type 2 diabetes and metabolic syndrome [43]. And there are also adverse outcomes in patient care [41,43]. When taking care of the health and well-being of nurses, managers should take into account all circumstances, not just the nurses’ preferences, and they should also inform them about the advantages and disadvantages of each form of work schedule.

In our study, no statistically significant differences were found in terms of gender, age, study status, childcare or parental care, which is in line with the results of research conducted by and Kelbiso et al. [10] and Suleiman et al. [33], but other studies have found differences in terms of gender [3,7], age and work experience [8,32].

In the BQNWL, nurses scored lowest on the questions about the adequacy of salary, job satisfaction and whether there are enough nurses in their workplace. In addition, a large number of nurses stated that they perform numerous non-nursing tasks and that the workload is high, but that they are able to provide good quality patient care. Although nurses stated that they are able to provide quality care, the insufficient number of staff in the departments leads to a high workload, which can also lead to missed nursing care. The level of salary, the number of nurses employed and the workload are factors that influence the evaluation of the quality of working life and the intention to change jobs and leave the profession [32].

Although two-thirds of the nurses had adequate WAI (excellent and good), 33.73% of the nurses had inadequate WAI (poor and moderate). This is a lower result than that obtained in the meta-analysis conducted by Romero Sanchez et al. [15], according to which the WAI was inadequate in 24.7% of nurses. In a previous study conducted in Croatia, 78.4% of nurses had an adequate WAI (excellent and good), while 21.6% had an inadequate WAI (poor and moderate) [44]. Nurses’ WA is related to both individual factors and factors related to the workplace. All these factors interact with each other and change throughout life and aging. Therefore, maintaining and supporting nurses’ health is crucial to ensuring their WA.

Although nurses reported a moderate QNWL, more than 60% of them had considered changing jobs in the past year. Nurses stated that they believe they could easily find a job with the same salary and benefits in another organization. Due to the shortage of nurses in almost all healthcare institutions, it is currently easy to change jobs, which could impact the number of nurses considering a job change. In our study, three-quarters of the nurses were younger than 50 years old, which may also affect the intention to change jobs and leave the nursing profession. In addition to QWL and WAI, the work of nurses in the Republic of Croatia has been influenced by numerous factors, such as the professionalization of nursing, socioeconomic factors, the COVID-19 pandemic and changes in the health sector. In the last 10 years, there has been a change in the educational structure of nurses; the number of nurses with a bachelor’s degree and the number of nurses with a graduate degree have increased. Unfortunately, for a large number of nurses, their level of education is not recognized in their current workplace, especially the recognition of graduate studies, which is a reason for dissatisfaction and can influence thoughts about changing jobs. Croatia also became a member of the European Union in 2013, making it easier for nurses to go to work in EU countries, as well as in other countries, which a large number of nurses have taken advantage of. Job offers that are provided can influence thoughts about changing jobs. The COVID-19 pandemic changed the way the work of nurses was organized, and during and immediately after the pandemic, some nurses changed jobs. There is also the development of the private healthcare sector (clinics, hospitals), which is opening new jobs and providing new opportunities for nurses for a secure workplace and perhaps easier working conditions, which may affect their intention to leave. Although no association between quality of work life and intention to change jobs was established in our study, the results of other studies show that quality of work life is negatively associated with turnover intention [25,32,45].

Nurses are the largest group of healthcare workers directly involved in the provision of healthcare. They are faced with an aging population and an increasing number of people with chronic and malignant diseases, which requires numerous nursing interventions, while at the same time, there is a shortage of nurses. In order to provide safe and quality care, have a sufficient number of satisfied nurses, retain nurses and create a favorable work environment for new nurses, managers need to monitor nurses’ quality of work life and work ability index [15].

In addition to monitoring QNWL and the WAI, it is necessary to systematically implement measures aimed at ensuring a sufficient number of nurses, ensuring flexible working arrangements, improving working conditions [46], taking into account family needs (childcare and provision of nurseries, provision of care for sick family members), ensuring conditions and space for rest during shifts and providing support for professional development and for nurses who are studying (days off for study, scholarships). The Ministry of Health and managers should find opportunities to increase the salaries of nurses and organize campaigns to increase the number of nursing graduates in order to have a sufficient number of employed nurses in the long term.

The work of nurses is characterized by a high level of stress. Therefore, it is important to pay attention to mental health, provide psychological counseling when needed, create a harmonious organizational atmosphere [4], teach nurses relaxation techniques and empower them to improve their resilience.

Nurse managers play a key role in building a positive and healthy work environment, which helps to reduce nurses’ turnover rate and improve their job satisfaction [4]. It is necessary to educate and empower nurse managers so that they acquire the necessary knowledge and skills to ensure a healthy work environment.

### Limitations

This study has several limitations. The questionnaire was distributed using the snowball method; nurses could leave the survey at any point during the research, and it is possible that nurses who were more interested in the research topic than others completed the questionnaires. Nurses completed the questionnaires themselves, which may influence the interpretation of the questions and possible answers.

The use of snowball sampling may affect the generalizability of the results since participants are recruited through referrals. It is also possible that participants who were more interested in the research preferred to forward the questionnaire to their colleagues with whom they share the same opinions.

A cross-sectional study was conducted, which prevents a generalization of the results, and it would be necessary to conduct longitudinal research to more accurately determine QWL, WAI and intention to leave the profession in more detail, as well as changes over a longer period of time.

Additionally, the study relied solely on self-reported measures without incorporating objective indicators, such as actual turnover rate, absenteeism or health data.

Furthermore, the study did not take into account the potential long-term effects of the COVID-19 pandemic on nurses’ work life quality, work ability, or career intentions, which may have influenced the results. Although data collection took place in May–June 2023, the Croatian healthcare system was still recovering from the COVID-19 pandemic. Residual organizational changes, heightened workload and prolonged psychosocial stress may have influenced nurses’ perceptions of work life quality, work ability and turnover intentions. As such, our findings should be interpreted with caution when extrapolating to pre-pandemic or completely post-pandemic conditions.

Finally, external factors such as the type of healthcare institution, regional differences and organizational characteristics were not controlled for, which may have influenced the results.

Since no association was found between QWL, WAI and intention to change jobs or leave the profession, future research should address these limitations by using randomized sampling methods, objective measures, longitudinal designs and qualitative approaches—such as semi-structured interviews or focus groups—to provide a more comprehensive understanding of the factors influencing nurses’ quality of work life, work ability, career intentions and complex personal, emotional and contextual motivations behind nurses’ considerations to leave their current job or the profession.

## 5. Conclusions

The aim of this study was to determine nurses’ quality of work life, work ability index and intention to leave the nursing profession and to examine the associations between nurses’ quality of work life, work ability index and intention to leave the nursing profession. Most nurses were found to have a moderate QWL and a good WAI, and a statistically significant positive correlation was found between the total BQNWL and the total WAI. Most nurses had considered changing jobs in the last 12 months, and more than a third of nurses had considered leaving the nursing profession. The study found no association between QWL, WAI and intention to change jobs or leave the profession. A large number of nurses are considering changing jobs and leaving the profession, even though they have a moderate QWL and an adequate WAI, which may be due to the ease of finding a new job due to the shortage of nurses and the desire for a better-paid job. To ensure the provision of necessary nursing interventions and a healthy working environment for nurses, it is necessary to regularly monitor QWL and WAI and implement interventions to ensure the highest quality of working life. Further research is needed on the connection between QWL and WIA and the intention to change jobs, as well as the reasons for thinking about changing jobs and leaving the profession. Furthermore, healthcare institutions should prioritize initiatives to improve working conditions by ensuring a sufficient number of nurses, reducing the workload, monitoring overtime work of nurses, promoting work–life balance, supporting career development, providing continuous education programs, providing scholarships for attending studies, ensuring recognition of completed education and improving nurses’ mental well-being. In this way, it will be possible to not only improve workforce retention but also ensure the continuous delivery of high-quality patient care.

## Figures and Tables

**Table 1:** Demographic characteristics.

		*n*	%
Gender	Male	53	10.64
	Female	443	88.96
	Don’t want to answer	2	0.4
Age (year)	21–30	116	23.3
	31–40	108	21.7
	41–50	132	26.5
	51–60	122	24.5
	61–65	20	4
Marital status	Single	88	17.7
	In a relationship	95	19.1
	Married	279	56
	Widowed	8	1.6
	Other	28	5.6
Children	Yes	322	64.7
	No	176	35.3
Age of the children	Preschool	56	-
	School	108	-
	18 years and older	158	-
Taking care of parents	Yes	182	36.5
	No	316	63.5
Studying	Yes	125	25.1
	No	373	74.9
	Undergraduate study	109	-
	Graduate study	16	-
Level of education	PhD	8	1.6
	Graduate study	164	32.9
	Undergraduate study	136	27.3
	Secondary nursing school	190	38.2
Job position	Rotation/shift work	224	45.0
	Morning work	145	29.1
	Shift leader	51	10.2
	On-call	18	3.6
	Other	60	12.0
Length of service	Years	0–48	Mean 20.34

**Table 2:** Total scores of nurses on BQNWL and WAI.

	Score Categories	*n*	%
Total QNWL score categories	low QNWL	8	1.8
	moderate QNWL	331	73.7
	high QNWL	110	24.5
	Total	449	100
Total WAI score categories	poor WAI	27	5.42
	moderate WAI	141	28.31
	good WA	218	43.78
	excellent WAI	112	22.49
	Total	498	100

**Table 3:** BQNWL total and domains.

	Possible Score	Actual Score	Mean	SD
Total scale score	42–252	93–240	165.26	23.72
Work life—home life	7–42	12–40	27.46	4.68
Work design	10–60	24–55	40.60	5.37
Work context	20–120	37–118	79.77	15.87
Work world	5–30	6–30	17.46	3.06

**Table 4:** Intention to change jobs/leave the nursing profession.

		*n*	%
Considered changing jobs in the last 12 months	Yes	277	61.1
	No	176	38.9
Considered leaving the nursing profession in the last 12 months	Yes	167	36.9
	No	286	63.1

**Table 5:** Presentation of intercorrelations between age (year of birth), length of service, BQWL subscales, total BQWL and total WAI.

		Age (Year of Birth)	Length of Service	Home Life Work Life	Work Design	Work Context	Work World	Total BQNWL	Total WAI
Age	Correlation Coefficient	1	−0.954 **	0.079	−0.013	−0.053	−0.066	−0.023	0.274 **
	*p*	.	0.000	0.097	0.791	0.265	0.168	0.633	0.000
	*n*	498	498	442	442	443	442	444	498
Length of Service	Correlation Coefficient		1	−0.078	0.016	0.062	0.076	0.031	−0.271 **
	*p*		.	0.103	0.738	0.191	0.112	0.516	0.000
	*n*		498	442	442	443	442	444	498
Home Life Work Life	Correlation Coefficient			1	0.329 **	0.404 **	0.188 **	0.558 **	0.315 **
	*p*			.	0.000	0.000	0.000	0.000	0.000
	*n*			442	441	442	441	442	442
Work Design	Correlation Coefficient				1	0.568 **	0.416 **	0.718 **	0.297 **
	*p*				.	0.000	0.000	0.000	0.000
	*n*				442	442	441	442	442
Work Context	Correlation Coefficient					1	0.507 **	0.952 **	0.343 **
	*p*					.	0.000	0.000	0.000
	*n*					443	442	443	443
Work World	Correlation Coefficient						1	0.598 **	0.214 **
	*p*						.	0.000	0.000
	*n*						442	442	442
Total BQNWL	Correlation Coefficient							1	0.386 **
	*p*							.	0.000
	*n*							444	444
Total WAI	Correlation Coefficient								1
	*p*								.
	*n*								498

Spearman’s rho, ** *p* < 0.01.

**Table 6:** Presentation of correlations between total BQWL, total WAI and considerations of changing jobs or leaving nursing profession.

		BQNWL Total	WAI Total	Considered Changing Jobs During Last 12 Months	Considered Leaving Nursing Profession During Last 12 Months
BQNWL total	Correlation Coefficient	1	0.386 **	0.018	−0.025
	*p*		0.000	0.721	0.616
	*n*	444	444	397	398
WAI total	Correlation Coefficient		1	0.058	0.070
	*p*			0.219	0.138
	*n*		498	449	450
Considered changing jobs during last 12 months	Correlation Coefficient			1	0.525 **
	*p*				0.000
	*n*			449	449
Considered leaving nursing profession during last 12 months	Correlation Coefficient				1
	*p*				
	*n*				450

Spearman’s rho, ** *p* < 0.01.

## Data Availability

The data in this study are available from the corresponding authors upon request. The data are not available to the public for confidentiality reasons.
